# The impact of human reservoir of malaria at a community-level on individual malaria occurrence in a low malaria transmission setting along the Thai-Myanmar border

**DOI:** 10.1186/1475-2875-9-143

**Published:** 2010-05-26

**Authors:** Saranath Lawpoolsri, Irwin F Chavez, Surapon Yimsamran, Supalap Puangsa-art, Nipon Thanyavanich, Wanchai Maneeboonyang, Wuthichai Chaimungkun, Pratap Singhasivanon, James H Maguire, Laura L Hungerford

**Affiliations:** 1 Department of Tropical Hygiene, Faculty of Tropical Medicine, Mahidol University, Bangkok, Thailand; 2 Department of Epidemiology and Preventive Medicine, University of Maryland School of Medicine, Baltimore, Maryland, USA

## Abstract

**Background:**

The probability of contracting malaria in a given individual is determined not only by the individual's characteristics, but also the ecological factors that characterize the level of human-vector contact in the population. Examination of the relationship between "individual" and "supra-individual" variables over time is important for understanding the local malaria epidemiology. This is essential for planning effective intervention strategies specifically for each location.

**Methods:**

A retrospective cohort study was conducted, which followed a community-cohort of about 3,500 residents in seven hamlets along the Thai-Myanmar border between 1999 and 2006. Potential malaria determinants measured at different levels (temporal variables, individual variables, and hamlet variables) were incorporated into multilevel models to estimate their effects on an individual's risk of malaria attack.

**Results:**

The monthly minimum temperature was significantly associated with the seasonal variation of malaria risk. An individual risk of malaria attack decreased by about 50% during the period that active surveillance was conducted; an additional 15% and 25% reduction of *Plasmodium falciparum *and *Plasmodium vivax *incidence, respectively, was observed after the use of artesunate-mefloquine combination therapy (ACT) for treatment of *P. falciparum*. Male children (age < 16 years old) were at highest risk of both *P. falciparum *and *P. vivax *attack. An increase in the hamlet's incidence of *P. falciparum *and *P. vivax *by 1 per 100 persons in a previous month resulted in 1.14 and 1.34 times increase in the risk of *P. falciparum *and *P. vivax*, respectively, among individuals in a particular hamlet.

**Conclusion:**

In a small area with low malaria transmission intensity, the variation in mosquito abundance is relatively similar across the residential areas; incidence of malaria between hamlets, which reflects the community level of human infectious reservoirs, is an important predictor for the malaria risk among individuals within these hamlets. Therefore, local malaria control strategies should focus on interventions that aim to reduce the gametocyte carriage in the population, such as early detection and treatment programmes and the use of ACT for *P. falciparum*.

## Background

Transmission of malaria is a dynamic process that involves human, vector, parasite, and environment. In addition, ecological factors, such as transmission intensity, and effectiveness of control efforts, also have a major impact on parasite transmission and malaria burden [1]. The risk of contracting disease for a given individual is greatly affected by factors at community-level, such as level of immunity or disease in the population, environmental conditions, culture, and general contact patterns among individuals in the same group or cluster [2,3].

Although the malaria risk factors at community-level, such as vegetation levels, immunity levels, and control programmes, have been widely studied in malaria research, these studies are generally interested in the aggregated outcomes, such as the overall incidence within specified clusters or areas; their results can be confounded by the variability among individuals within clusters and may not reflect the actual risk in an individual [3,4]. On the other hand, an individual-level study that does not take into account the contextual components may miss important disease determinants [5]. While the relations between individual and ecological determinants are of particular interest for understanding malaria epidemiology, it is important to simultaneously incorporate factors defined at multiple levels in epidemiological analysis.

The relative importance of specific individual-and community-level factors is likely to differ in areas of hyper-and hypo-endemicity. For example, in high malaria transmission areas, individual immune status, leading to asymptomatic infection in adults, and competent and efficient malaria vectors play an important role in malaria transmission and malaria clinical episodes [1,6]. Therefore, environmental conditions, such as altitude, rainfall, humidity, and temperature, which affect the vector density, could have a substantial impact on malaria transmission in these hyper-endemic areas [1]. On the other hand, in low malaria transmission areas, mosquito vectors are usually less efficient and a large fraction of population is susceptible to the disease. Not only optimal environmental conditions, spatially and temporally, favoring for vector are required for the transmission, but also density of infectious individuals in the population may become a significant determinant for sustaining the transmission in the areas [7-10]. However, studies that examine the effect of the changes in density of human reservoir at community level on the individual risk of malaria have not been established in malaria low-transmission areas.

The objective of the present study was to examine the effect of potential malaria risk factors at both community-level and individual-level factors on individual malaria risk during an 8-year period in a small geographical area along the Thai-Myanmar border.

## Methods

### Study site and study population

**Figure 1:**
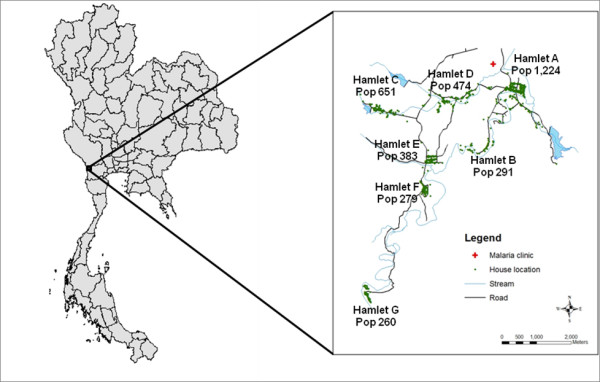
**Study area and study population**. Study area consists of seven hamlets in Suanphung district, Ratchaburi, Thailand.

The study site is located in a mountainous area in Suanphung district, western border of Thailand. The 6 × 8 km^2 ^study area consists of approximately 500 households in seven hamlets with a human population of about 3,500. Hamlets are interconnected by narrow roads or dirt roads. Houses are clustered close to a stream that runs through all the hamlets (Figure 1). Climate is typical for a tropical region; the mean mid-day temperature is 26°c (range 18°c-31°c); mean humidity is 79% (range from 76% to 81%); and average annual rainfall is 1.3 meters, with a peak in the rainy season from May to August. *Anopheles minimus*, *Anopheles maculatus*, and *Anopheles dirus*, night-time and outdoor-biting mosquitoes, are the main malaria vectors.

*Plasmodium falciparum *and *Plasmodium vivax *are the most common cause of malaria in the area. Although this area is classified as a low malaria transmission area, malaria accounted for about 10% of all fever cases presented at a local malaria clinic [11]. The early detection and prompt treatment through passive surveillance was the main malaria control program in the area, since the malaria clinic was established in 1997. An additional active surveillance program that sampled more than half of the population in all hamlets each time has been conducted every three to four months since 2003. Asymptomatic infection was occasionally detected by a routine active surveillance programme during the early phase of the programme, about 1% of samples in 2003. Asymptomatic infection was rarely detected since year 2005. A single dose of mefloquine 750 mg and primaquine 30 mg, adjusting for age, was used as the first-line drug for treatment of uncomplicated *P. falciparum *malaria until the year 2005; because of the increased mefloquine resistance (efficacy less than 85%), the standard regimen was changed to artesunate-mefloquine combination therapy (mefloquine 750 mg. and artesunate 300 mg, followed by mefloquine 500 mg at 6 hour intervals on day 1). The standard regimen (chloroquine 1500 mg. base over 3 days combined with primaquine 15 mg. daily for 14 days) for treatment of *P. vivax *has not been changed over the time. There is only one local malaria clinic in the area. People rarely seek treatment outside; thus almost all clinical malaria cases can be detected at the malaria clinic. All malaria cases were based on microscopic diagnosis.

### Data collection

#### Census and geographical data

Detailed demography data of all residents were obtained through active surveillance surveys conducted by staff of the malaria clinic in 1998, 2001, and again every three months from 2003 to 2006. A linear trend of population growth was applied to year 2000 and year 2002, when detailed census was not available. These census data were used to form a dynamic cohort of 4,380 residents between 1999 and 2006.

Geographic locations of houses, roads, stream, and the malaria clinic were obtained from a geographic survey in 2005 using Garmin III+ handheld Global Positioning System (GPS) receivers with accuracy within 15 meters. Coordinates of these locations and their attribute data of household members and individual malaria occurrence were entered into the ArcGIS 9.0 software to create a geo-referenced database and a map for the study area (Figure 1). Among the 4,380 individuals recorded in the census, 3,562 individuals who had geographical location data were included in the dynamic cohort for the study. People with missing location data were likely those who came from other areas with lower risk of malaria infection in Thailand and had probably stayed in the area temporarily for work, according to their demographical profiles. Although illegal migration from neighbouring country is possible in this area, the number of migrants is expected to be small, because of the geographical limitation for transportation, and a close monitoring by military troops. The study was approved by the Ethical Committee of the Faculty of Tropical Medicine at Mahidol University, Thailand, and the Institutional Review Board of the University of Maryland, School of Medicine.

#### Malaria occurrence

Information regarding individual malaria clinical episodes was obtained from records of the Rajanagarindra Tropical Disease International Center (RTIC), the local malaria clinic, between 1999 and 2006. Additional malaria cases, both symptomatic and asymptomatic, were obtained from the routine active surveillance, which began in 2003. All malaria diagnoses were microscopically confirmed by well-trained staff at the clinic. Recrudescence and relapse episodes were defined as repeat *P. falciparum *malaria attacks within 30 consecutive days or repeat *P. vivax *attacks within 90 days after the initial episode, respectively. Recrudescence and relapse episodes were considered as a part of the initial episode and were not counted as new malaria infections.

#### Temporal (within-individual) variables

Mean monthly minimum/maximum temperature, mean monthly humidity, and monthly amount of rainfall, recorded daily at the malaria clinic, were used to determine the seasonality effect on the temporal change of individual malaria risk. The effect of the routine active surveillance and the effect of artemisinin-mefloquine combination therapy were applied to the period beginning in years 2003 and 2005, respectively. Residual spraying programme was the main vector control in the area, which conducted twice a year in February and August, months after the observed peak malaria incidence. The impact of the vector control is expected to be less, because of the delayed timing of the implementation. In addition, the pattern of vector control programmes did not change over the study period. Therefore, variables regarding vector control were not included in the study.

#### Individual-level variables

Individual demographic characteristics, including age, gender, and an age-gender interaction, were studied. The age variable was categorized into three groups (0-15, 16-50, and 51+ years old), which likely reflects the difference in behavioural exposure to vector. Although the distance from individual house to a stream was measured at household level, the differences in the distance were relatively large among individuals within a hamlet. Therefore, the proximity to a stream was considered and included in the analysis as a variable at the individual-level.

#### Hamlet-level variables

Hamlet characteristics analysed in the study included: proximity to the malaria clinic, previous incidence of malaria in the hamlet, and landscape features. Distance from individual house to the malaria clinic was more likely to be similar among individuals within the same hamlet, but largely different among individuals in other hamlets. This could represent the difference in access to treatment among hamlets. In an area such as this one, where malaria incidence is geographically clustered, the individual risk of contracting malaria disease could depend on the level of infectiousness in neighboring people. Since the malaria parasite usually takes about 30-40 days from mosquito blood feeding on an infectious individual until successful transmission of the parasite and the resulting clinical symptoms in another individual [12], a one-month lag of the malaria incidence within a hamlet was used to represent the density of human reservoirs in the community that contribute to malaria burden for a particular case. Other lags (0-2 months) were used to assess general clustering that could be due to other factors.

**Figure 2:**
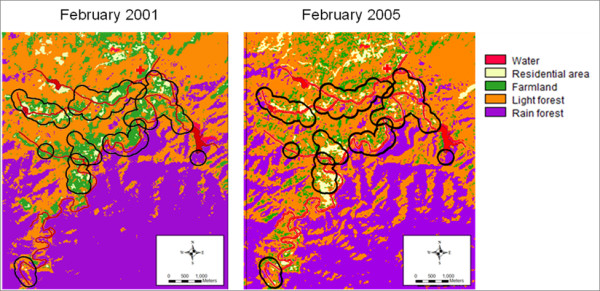
**Landscape features of the study area**. Landscape feature pattern and 250-meter buffers of the seven hamlets, Tanousri subdistrict, Ratchaburi, Thailand. Supervised classification of LandSat 5 Satellite images taken in February 2001 and February 2005.

To identify landscape feature characteristics of each hamlet, two 7-band LandSat 5 Satellite images of the study area with a spatial resolution of 30 × 30 meters taken in February 2001 and February 2005 were obtained and processed. The satellite images were classified into five different landscape features: water, residential areas, farmland, light forest, and dense forest, using a supervised classification method with ground truthing in 73 selected points conducted in 2007. Farmlands were characterized by the presence of any agriculture. Light forests were defined as areas covered by trees that are accessible to people. Forest areas that were difficult to enter, such as on a mountain top, were classified as dense forest. A map with 250-meter radius buffer of houses in each hamlet was created and then overlaid on the classified landscape feature maps (Figure 2). A buffer of 250 meters was chosen based on the maximum buffer radius that showed a difference between malaria case density measured within a buffer of houses with malaria cases and that measured within a buffer of malaria-free houses during rainy season each year. Total area in square meters and percentage of areas covered by each landscape feature type were calculated for each hamlet buffer (Table 1). The percentages of landscape feature types obtained from the 2001 and 2005 maps were then used to represent the landscape features during the period of 1999-2002 and 2003-2006, respectively.

**Table 1:** Landscape characteristics of the study area in 2001 and 2005 by hamlet.

	Hamlet													
	
	A		B		C		D		E		F		G	
Total area (m^2^)	2552500		1092500		1885625		1182500		973125		825000		526250	
**Landscape features**														
Year of Images	2001	2005	2001	2005	2001	2005	2001	2005	2001	2005	2001	2005	2001	2005
Water features,%	0	0	0	0	0	0	1	0	0	0	0	0	0	0
Residential area,%	6	7	2	4	8	6	18	16	6	18	9	31	1	6
Farmland,%	33	11	35	15	33	20	35	18	50	21	51	17	1	14
Light forest,%	47	67	31	55	57	68	45	62	41	55	39	50	38	56
Dense forest,%	13	15	32	26	1	6	1	4	4	6	1	2	60	24

### Data analysis

Individual occurrences of both *P. falciparum *and *P. vivax *were determined for each month during the period that the people appeared in the census; individual monthly malaria occurrences were then used as repeated outcome measures that nested within each individual. This nested data structure resulted in dependency of outcome and the similarity of characteristics among repeated measures within the same individual or among individuals within the same hamlet, which may lead to an artificially small variance in the population [3,13,14]. Therefore, a multilevel analytical approach using a generalized linear mixed model (GLMM) was performed to simultaneously analyse the data at different levels on an individual outcome, while accounting for the non-independence of within-individual and within-hamlet observations, and allowing the baseline effects of variables to vary across individual and hamlet, i.e., the random effects were hamlet and individual intercepts [2-4,14,15].

Univariate analysis was initially performed to examine the association between each explanatory variable and individual malaria risk. Significant variables at 0.10 level were considered for inclusion in the multivariate analysis. Because of the collinearity among different categories of landscape feature types or between two temperature variables (minimum and maximum), only one landscape feature type and one temperature variable with the strongest significance level were included in the multivariate model. The variables with the strongest univariate relationships were carried forward. Potential biologically plausible intra-level interaction and cross-level interactions were identified. An interaction was considered important at 0.10 level.

Multivariate generalized linear mixed models were then fitted to identify the most parsimonious model using a backward elimination technique. Only mean monthly humidity and monthly amount of rainfall lagged zero to two months, were not significant in the univariate analysis. Therefore, all other significant variables and interactions from the univariate analysis were included in the full model; variables that had the least significance and did not affect the main variable were progressively removed from the full model. The analysis was performed separately for *P. falciparum *and *P. vivax *using SAS (PROC GLMMIX) version 9.1; *P. falciparum *and *P. vivax *mixed infection episodes were included in both models. Adjusted odds ratios (ORs) and 95% confidence intervals (CIs) were then calculated for all potential risk factors that remain significant after being introduced into the multivariate model.

## Results

### Demography and overall malaria distribution

Demographic characteristics of the study population by hamlet are illustrated in Table 2. Population size varied across hamlets. The number of residents in hamlet A accounted for about one-third of the overall population. There were no significant differences in age or gender distributions among hamlets. Males and children aged less than 16 years old accounted for about half of the population in each hamlet.

**Table 2:** Demographic and landscape characteristics of the study population by hamlet.

	Hamlet						
	
	A	B	C	D	E	F	G
Total population, N	1224	291	651	474	383	279	260
Gender							
Male, %	52	54	52	57	51	47	59
Female, %	48	46	48	43	49	53	41
Age group							
0-15,%	48	61	55	54	51	56	48
16-50,%	46	35	39	40	41	46	45
51+,%	6	4	6	6	8	8	7
Range of monthly malaria incidence (per 100 persons)							
*P. falciparum*	0-4.24	0-6.84	0-4.21	0-4.06	0-6.56	0-4.91	0-10.57
*P. vivax*	0-1.94	0-3.77	0-1.69	0-2.87	0-4.06	0-2.68	0-4.65

The incidence of both *P. falciparum *and *P. vivax *varied across place and time (Table 2). Within the hamlets, the peak monthly incidence (per 100 persons) varied from 4.06 (hamlet D) to 10.57 (hamlet G) for *P. falciparum*, and from 1.69 (Hamlet C) to 4.65 (hamlet G) for *P. vivax*.

### Temporal risk factors

Individual risk of contracting both *P. falciparum *and *P. vivax *was significantly associated with ambient temperature, the deployment of routine active surveillance, and the use of artesunate-mefloquine combination therapy (ACT) (Table 3). However, the effect of temperature was stronger for *P. falciparum *than for *P. vivax *(OR 1.27 vs. 1.05). The risk of *P. falciparum *and *P. vivax *reduced by 40% and 60%, respectively, after active surveillance had been implemented. Although the ACT was applied only for treatment of *P. falciparum*, a substantial reduction of *P. vivax *risk was also found during the period of ACT use.

**Table 3:** Adjusted odds ratios (ORs) and 95% confidence intervals (CI) for potential malaria risk factors by *Plasmodium *species, longitudinal data from 1999 to 2006.

	*P. falciparum*		*P. vivax*	
	
	OR	95%CI	OR	95%CI
**Temporal Factors**				
Mean minimum temperature (1°c)	1.27	1.23-1.31	1.05	1.02-1.09
Control measures				
Passive surveillance only		1.00		1.00
Presence of Active surveillance	0.35	0.25-0.50	0.49	0.41-0.58
Active surveillance + ACT use for *P. falciparum*	0.21	0.15-0.30	0.22	0.17-0.28
**Individual-level**				
Females				
0-15	1.00		1.00	
16-50	0.51	0.44-0.59	0.22	0.17-0.29
51+	0.41	0.29-0.59	0.15	0.07-0.31
Males				
0-15	1.00		1.00	
16-50	0.68	0.61-0.75	0.28	0.23-0.33
51+	0.50	0.39-0.64	0.23	0.14-0.37
**Hamlet-level**				
Light forest cover area (1% increase)	1.03	1.01-1.05		
Distance to a clinic (1 km increase)	1.31	1.15-1.49	1.08	0.98-1.18
1-month lag incidence of *P. falciparum/P. vivax *per 100 persons	1.14	1.11-1.17	1.34	1.23-1.45

**Covariance of random effects**	Var	SE	Var	SE
Hamlet	0.152	0.143	0.063	0.046
Residual	0.983	0.003	1.009	0.003

### Individual-level risk factors

The risk of both *P. falciparum *and *P. vivax *attack varied with individual age and gender. Male children had the highest risk of contracting malaria, as compared to other age and gender groups. In general, the malaria risk decreased with age for both males and females; the age effect was stronger among females than males. Distance from individual house to a stream was not associated with malaria risk in this population.

### Hamlet-level risk factors

The risk of contracting *P. falciparum *and *P. vivax *for an individual was significantly affected by the level of *P. falciparum *and *P. vivax *incidence within the hamlet to which the individual belongs (Table 3). An increase in the one-month lag hamlet incidence of *P. falciparum *and *P. vivax *by only 1 per 100 persons resulted in 1.14 and 1.34 times increase the risk of malaria among people in the particular hamlet. Other time lags were not statistically associated with malaria risk. The association between distance to the malaria clinic and malaria risk was significant only for *P. falciparum*. The risk of *P. falciparum *infection increased by 1.13 times for every one kilometer increase in the distance from house to the malaria clinic.

Although the distribution of vegetation types varied across hamlets and time, more than half of each hamlet was covered by forest (Table 1). Since light forest is an important mosquito habitat and showed statistical significance in the univariate analysis, the percentage of light forest cover area was selected for the multivariate models. However, the multivariate analysis revealed that light forest cover was a significant risk factor only for *P. falciparum*. The risk of *P. falciparum *attack among people in a hamlet increased by 1.03 for every 1% increase in the hamlet's light forest cover area (Table 3).

None of the cross-level interactions examined in the analysis was significant. The effect of the one-month lag hamlet incidence on an individual malaria risk did not vary significantly by the distance from house to stream, presence of the active surveillance, and individual age-gender profile. The remaining random effect components revealed that the baseline malaria risk varied among hamlets even after controlling for the variables in the model; this between-hamlet variation was larger regarding the risk for *P. falciparum *than for *P. vivax*.

## Discussion

Malaria occurrences are not randomly distributed across space and time [16]. This variation is likely influenced by the combined effect of factors that characterize individuals and areas [2]. Findings of the study show that factors at both the community-level and individual level are important in understanding the variation of malaria risk among individuals. In addition, temporal changes in the community factors, e.g., seasonal variation and control intervention programmes, can have a major impact on individual malaria outcome.

The relationship of malaria incidence with monthly minimum temperature rather than rainfall may be unique to this wet, higher altitude area. While the optimal temperature for the development of both vector and parasite is between 25°C and 30°C, the lower temperature during dry season may jeopardize the transmission capability of mosquitoes [17-19]. Since *P. vivax *is more tolerant at a relatively low temperature condition, the effect of temperature would be expected to be less for *P. vivax *than *P. falciparum*, which is consistent with the study findings. This may provide opportunities for new strategies that target interventions to eliminate transmission during these periods when temperature favors success. It may also raise concerns about future failure of control programmes in such regions if warming climate occurs even without changes in rainfall.

Malaria control interventions were associated with a substantial impact on the inter-annual reduction in malaria risk in this population, after adjusting for the temperature effect. Routine active surveillance allows detection of asymptomatic cases, which are the important infectious reservoirs of malaria [20-22]. In addition, symptomatic patients can be detected and treated earlier during active surveillance than when passive surveillance is operating alone [18]. The resulting of reduction in both the number of reservoir hosts and in an individual's infectious period could both decrease infection pressure in a hamlet.

The finding of the effectiveness of artemisinin-based combination therapy (ACT) on *P. falciparum *occurrence is consistent with research conducted in other areas [23-25]. Artemisinin has been shown to be effective in killing both the asexual stages and the immature sexual stages of *P. falciparum *parasites, which consequentially prevents onward transmission [26-29]. Although ACT was used only for treatment of *P. falciparum*, a substantial reduction in *P. vivax *incidence was also observed during the ACT use period; this could be possibly explained by the additional effect of artesunate in killing asexual *P. vivax *in patients who initially had mixed infection. Mixed infection is likely to be undetected using microscopy [30]. In Thailand, using molecular techniques, mixed *P. falciparum *and *P. vivax *infections have been reported in about 60% of clinical malaria patients, and when it presents, *P. falciparum *generally dominates [30]. Although ACT has no reported impact on eliminating *P. vivax *hypnozoites, the liver stage parasite that causes relapse, a clinical trial conducted in Thailand has shown that about 50% of *P. vivax *patients who received either 5- or 7-day artesunate treatment remained parasite-free at day 28 after the treatment [31]. However, the effect of ACT on reducing subsequent *P.vivax *reappearance among submicroscopic mixed infection patients is still unclear.

The effect of age and gender on malaria incidence in low transmission settings varies across areas [21,22,32,33]. Unlike areas with high malaria transmission, an increased risk of malaria attack among children in malaria low transmission areas could be easily attributable to their lack of development of immunity, since exposure and clinical episodes are uncommon in all age groups [1,32]. The pattern of age-gender distribution of malaria risk may, in part, be due to behavioural exposure to vector or to other unmeasured variables, such as intrinsic differences in immune function or hormonal response among different age groups and genders [34,35]. Future work to explore this pattern, including immunological tests, is needed in hypo-endemic areas.

In this study, the risk of malaria attack was not significantly different for individuals living at variable locations relative to a stream. Although an association between topographical characteristics and malaria incidence has been reported in many areas, these observations are generally where there are large variations in attributes of house locations [21,36-38]. For example, an increased risk of malaria was found to be associated with houses located farther than 500 or 1,000 meters from a river [21,36,37]. However, in this study area, the mean distance from an individual house to a stream was about 200 meters with a range of 200 to 500 meters. This relatively small variation may mean that vector abundance is similar among houses. The hamlet's light forest cover area had a marginal effect on the risk of *P. falciparum *attack but no effect on the risk of *P. vivax *attack. There are differences in the preferred habitats of Anopheles vector species in Thailand. *An. minimus*, a predominant vector in Thailand, is commonly found close to stream margins in forested and foothill areas [39-41]. However, the association between hamlets' light forest area and malaria risk was still uncertain due to limited number of landscape data for comparison at hamlet-level. Future research with wider spatial and temporal coverage is needed to understand which environments might favor transmission.

Malaria transmission directly depends on the presence of a human reservoir as well as the level of vector-human contact [1,42]. While the factors most commonly found to be associated with vector abundance were relatively stable across the area, changes in the number of infectious human reservoirs in the community could potentially affect transmission probabilities for individuals in the population [43,44]. For high transmission areas, stable areas of higher and lower incidence have been associated with spatially identifiable transmission and control factors. However, in low transmission areas where the majority of cases are quickly treated, other factors, such as immigrant cases or individual risk behaviours might be more important. In this study, the addition of one human reservoir per 100 persons in a hamlet resulted in 1.14 and 1.34 times higher risk for contracting *P. falciparum *and *P. vivax*, respectively, among individuals who lived in the hamlet. Spatial variation in level of gametocyte carriage in the population may have a major role in defining risk for malaria transmission in low endemicity areas.

Gametocyte carriers are vital for sustaining the disease in a population. However, number of humans available to infect mosquitoes may not be a limiting factor in high endemic regions. Interventions that have a strong community-level effect, particularly early detection and treatment to decrease number and duration of gametocyte carriage may have a more powerful effect on reducing malaria transmission in hypoendemic regions [18,23]. The study findings showed that people who lived far from the malaria clinic were more likely to have *P. falciparum *attacks than those who lived closer. Distance to the malaria clinic may reflect how quickly people receive treatment. Those who live farther may take longer time to seek treatment, which consequently increases the infectious duration. Although the Euclidean distance used in the study was not accounted for the actual route and terrain to the clinic, the pattern of main roads and terrain features of the area suggests that the Euclidean distance and via route distance are likely to be proportionately similar.

Results of this study may be subject to misclassification bias. The incidence of malaria in a previous month was used as a proxy for the level of infectious individuals in each hamlet. For malaria infection, especially *P. falciparum*, infected patients do not always represent an infectious population. While this would bias estimates of incidence for each hamlet, the OR's would be biased toward the null (underestimated) unless the rates of infectious to infected individuals varied systematically between hamlets, which could possibly occur if the pattern of treatment and clearance of parasites differed across hamlets. Misclassification of landscape features was also possible because of the limited resolution of LandSat images. Additionally, land use changes were occurring over this period in several of hamlets, which can have variable effects on vector dynamics. However, characterizing spatial attributes may be less critical for a region with widespread water and vector habitat availability.

Although the one-month lag time used for hamlet incidence was based on the time from parasite uptake from one person until clinical symptoms appear in another individual, this lag time did not account for different durations of infectiousness and the infectivity dynamic among individuals. An individual's infectious period is usually less than one week. However, some individuals may remain infectious up to a month, even after treatment [44,45]. In addition, recrudescence may prolong the duration of infectiousness; and relapse may result in multiple episodes of infectious period. Individuals who had a long infectious period could continue to infect mosquitoes and be a source for the disease in their hamlets in the next two or three months. However, in this study area, short duration from clinical attack to treatment and the low number of asymptomatic cases found during active surveillance suggest that a prolong individual's duration of infectiousness may be less likely, with an exception in 2003 and 2004 when recrudescence was observed in about 10% to 15% of all *P. falciparum *malaria cases, due to mefloquine resistance. Additionally, previous higher incidence in a hamlet may simply be a proxy for a higher risk in location where malaria is generally found. Findings from the preliminary analysis showed that the locations with highest incidence moved over time in this study area. Further, the effect of hamlet incidence became non-significant in the sensitivity analysis using different lag times (zero and two months lag).

To study the epidemiology of malaria, one must consider the relationship among factors that characterize time, person, and place. In this study, potential malaria risk factors at temporal-level, individual-level, and community-level were simultaneously examined. The 8-year longitudinal data used in the study allowed us to determine the effect of temporal and spatial changes over time. Unlike ecological studies where inferences are drawn on the basis of aggregated outcomes for a group, the individually specified, hierarchical outcomes used in this study provide more valid inferences regarding malaria. Results of this study provide new insight into the malaria epidemiology in a low malaria transmission area, where malaria elimination is feasible. Moreover, similar patterns of *P. falciparum *and *P. vivax *risk factors found in the study suggest that a uniform intervention could have an effect on both *P. falciparum *and *P. vivax *incidence. However, further research is needed to investigate the effects of ACT on *P. vivax *among individuals with submicroscopic mixed infection.

## Conclusion

The incidence within a hamlet can be a good predictor for individual risk of malaria in a subsequent month; therefore, a targeted malaria monitoring and effective surveillance system could be keys for timely implementation of malaria control programmes. Also, effective intervention programmes that aim to reduce gametocyte carriage could have a major impact on interrupting the malaria transmission in the area.

## Competing interests

The authors declare that they have no competing interests.

## Authors' contributions

SL, PS, JHM, and LLH conceived and designed the study. SL analysed the data. IFC, SY, SP, NT, WC, and WM managed the data set. SL, LLH, and JHM wrote the paper. All authors read and approved the final manuscript.
